# Epidemiological update on spatial and space–time clusters of African swine fever in the Philippines

**DOI:** 10.3389/fvets.2026.1888714

**Published:** 2026-09-29

**Authors:** Jesper Chia-Hui Hsu, Rachel A. Schambow, John M. Humphreys, Maximino Montenegro, Ruth Miclat-Sonaco, Andres M. Perez

**Affiliations:** 1 Center for Animal Health and Food Safety, College of Veterinary Medicine, University of Minnesota, Minneapolis, MN, United States; 2 U.S. Department of Agriculture, Agricultural Research Service, National Bio and Agro-Defense Facility, Manhattan, KS, United States; 3 Pig Improvement Company (PIC) Philippines, Pasig, Philippines; 4 National Livestock Program, Department of Agriculture, Office of the Undersecretary for Livestock, Quezon City, Philippines

**Keywords:** African swine fever, Asia-Pacific, disease dynamic, Genotype II, spatial epidemiology

## Abstract

African swine fever (ASF), particularly the highly contagious Genotype II strain, is a transboundary viral disease causing up to 100% mortality in swine and threatening food security across the Asia-Pacific region. Since 2019, control strategies in the Philippines have relied on depopulation and strict administrative zoning to limit contiguous, land-based spread. However, the long-term effectiveness of these approaches within the country’s fragmented archipelagic landscape remains poorly understood, complicating evidence-based resource allocation and preparedness for regional vaccination campaigns. Here we show that the endemic trajectory of ASF was highly non-uniform across the three main island groups of the Philippines from October 2022 to October 2024. Spatiotemporal analysis of a national surveillance dataset comprising 8,213 barangay-level events revealed twelve statistically significant retrospective space–time clusters—with radii up to 183.2 kilometers—and highly asynchronous regional epidemic waves across Luzon, the Visayas, and Mindanao. Although crude case volumes were highest in Luzon (52.10%), followed by the Visayas (31.69%) and Mindanao (16.21%), accounting for swine population density reshaped the spatial distribution. Spatial mapping and a Getis-Ord Gi* hotspot analysis consistently identified the highest burden of reported ASF events within Regions IV-A, IV-B, and VI, specifically peaking on Mindoro Island and in the Western Visayas. Together, these findings demonstrate that maritime borders fail to provide natural biosecurity isolation against transboundary viral transmission. Consequently, region-specific interventions and resources must be dynamically allocated to address this heterogeneous spatial burden. These spatiotemporal results provide the evidence-based framework necessary to transition toward targeted sub-national surveillance and to systematically evaluate the epidemiological impact and guide live-attenuated vaccine implementation in the Philippines and analogous archipelagic settings throughout the Asia-Pacific.

## Introduction

African swine fever (ASF) is a highly contagious swine transboundary infectious disease affecting domestic and wild pigs. The mortality rate of ASF is high, often reaching 100% in peracute or acute forms (1, 2). It is an important transboundary animal disease that is reportable to the World Organisation for Animal Health (WOAH, formerly known as OIE). Although it is not a zoonotic disease, it has a significant impact on the global swine industry. Since the 2018 outbreak in China, ASF has emerged as a significant threat across Southeast Asia (3). Between 2018 and 2025, more than 20 countries in the Asia-Pacific region reported local outbreaks in either domestic pigs or wild boars, according to the Food and Agriculture Organization (FAO) (4).

In the Philippines, ASF Genotype II strain was first detected in July 2019 (5), with the initial outbreak in Luzon Island. Since then, the Bureau of Animal Industry (BAI) has implemented several nationwide programs to control and manage ASF. These governmental initiatives are designed to reduce disease transmission and assist in repopulating swine herds in both commercial and backyard farms (6). Key measures include establishing a National Zoning and Movement plan, carrying out depopulation campaigns (7), and launching the National ASF Prevention and Control Programme (NASFPCP) (6, 8). The ultimate goal of these coordinated efforts and network is to achieve sustained control of the disease and eventually eradicate it. The Philippine Statistics Authority (PSA) reports that after a significant drop in the volume and annual growth rate of hog production in 2021 (a 17.8% decrease compared to previous year, 2020), the national swine population has begun to show a mild recovery. This rebound is evidenced by positive growth rates of 4.5% in 2022 and 3.3% in 2023 (9). However, this trend does not indicate the eradication of ASF, which remains a long-term threat to the country. As of 2025, outbreaks have occurred in 76 of the Philippines’ 82 provinces, with six provinces reporting active cases (10, 11). A recurring challenge is that outbreaks often resurface in provinces after herd depopulation and the subsequent reintroduction of new pigs, indicating a persistent transmission pattern in multiple islands and regions (12–14).

Given the upcoming consideration of ASF vaccination under the national prevention program, with vaccine procurement and distribution planned for several priority regions (15, 16), it is crucial to establish a robust baseline of the current ASF endemic situation. This baseline is essential for evaluating the future impact of interventions, as vaccination is expected to alter disease dynamics, shift herd immunity thresholds, and modify key transmission parameters such as the basic reproduction number (17). To construct this baseline, this study leverages longitudinal surveillance data from the Bureau of Animal Industry’s ASF Bulletin, which provides regular monthly reports on ASF outbreaks across the Philippines administrative units. While this dataset represents the most comprehensive and best available national records, it is subject to inherent field-level constraints, including potential underreporting, diagnostic delays, and spatial variations in surveillance intensity. Acknowledging these uncertainties, the granularity of the dataset still allows for spatial and temporal mapping, effectively turning localized administrative records into a roadmap of ASF dynamics. A previous study analyzed the spatiotemporal dynamics of ASF in the Philippines from 2019 to 2022 (12). Building on that work, our study updates the analysis using surveillance data from October 2022 to October 2024, a critical period during which ASF became endemic in many administrative regions. By characterizing these recent patterns of ASF occurrence, our study provides an updated epidemiological overview. This information can inform targeted prevention and control strategies and serve as a crucial reference for evaluating the impact of forthcoming vaccination campaigns.

## Methods

The African swine fever surveillance dataset was collected from October 5, 2022, to October 18, 2024. Samples were collected at the barangay level and diagnosed at the Regional Animal Disease Diagnostic Laboratory (RADDL), institutions whose laboratory services meet international WOAH standards. The dataset included geographic and administrative information such as island group (Luzon, Mindanao, and Visayas), region, province, city/municipality, barangay, barangay centroid coordinates (longitude and latitude), and the date of the latest report from the local government. These raw data were compiled and summarized at the national level by the Bureau of Animal Industry, Government of the Philippines. In this study, an epidemiological “case” or “report” is defined at the barangay level in the Philippines; a single report signifies the confirmed presence of at least one ASF outbreak event within that specific barangay during the study period, rather than individual animal infections. To characterize host demographics, swine population density data were obtained from the Food and Agriculture Organization (FAO) Gridded Livestock of the World (GLW4, 2020), which provides a 10 km resolution raster GIS layer. To evaluate the spatiotemporal burden of ASF, the standardized ASF outbreak reporting burden was calculated across defined administrative units (year of 2022). This metric estimates the probability of an epidemiological unit experiencing an event over a specific timeframe (18, 19). It was calculated by dividing the total unique ASF-positive barangay reports recorded in the study period by the baseline swine population at risk within the corresponding administrative boundary, aggregated from the FAO GLW4 dataset.

Exploratory analyses were performed to assess spatial clustering, temporal patterns at the island and regional levels, and space–time aggregation patterns. Spatial statistics and cluster mapping were conducted using the ArcGIS software (Pro 3.1.0; ESRI Inc., Redlands, CA, USA) and R. Specifically, the R packages **sf** (20), **terra** (21), and **stars** (20) were utilized for spatial data management, geodata for administrative boundaries, and **spdep** for autocorrelation testing (22). Hotspot detection was performed using the Getis-Ord Gi* statistic method, which calculates a *Z*-score for each spatial feature to determine the degree of clustering. Features with high positive *z*-scores represent statistically significant hot spots, where high values cluster spatially; conversely, features with low negative *z*-scores represent statistically significant cold spots, where low values cluster spatially.

Data preprocessing and temporal aggregation were conducted in R version 4.4.3, using the **tidyverse** package for data cleaning and exploratory analysis. Space–time cluster detection was performed in SaTScan version 10.3.2, employing a retrospective space–time analysis with the Space–Time Permutation model. Parameter settings included a maximum spatial window of 50% of the population at risk, a temporal aggregation length of 3 months, a maximum temporal cluster size of 50% of the study period, a circular window shape, and 999 Monte Carlo replications to identify clusters with a significantly elevated number of ASF cases.

## Results

A total of 8,213 ASF events were recorded at the barangay level during the study period. The geographical distribution of these outbreaks was not uniform, as evidenced by the disproportionate impact across major three island groups. Luzon island accounted for the majority of the dataset with 52.10% (*n* = 4,279) of the total volume, followed by the Visayas at 31.69% (*n* = 2,603), while Mindanao represented the smallest portion at 16.21% (*n* = 1,331). Further details regarding regional and provincial distributions are provided in Supplementary Table S1, which presents the outbreaks in both absolute counts and percentages; notably, Region VI recorded the highest number of events with 2,172 reports, representing approximately 26.45% of the total. Following this, a few other regions also reported a substantial number of outbreaks, each contributing more than 10% of the total: Region IV-B (14.68%), Region IV-A (13.80%), and Region XII (11.06%). Together, these four regions (IV-A, IV-B, VI, XII) accounted for nearly two-thirds of all recorded ASF events, highlighting a geographical concentration of the disease during the study period.

To visualize the spatial distribution of recorded ASF cases, a province-level choropleth map was generated by aggregating barangay-level data across 82 provinces (Figure 1B). The analysis reveals that certain provinces were disproportionately affected; Oriental Mindoro (Region IV-B) recorded the highest number of affected barangays (*n* = 807), followed by Capiz (Region VI, *n* = 727), North Cotabato (Region XII, *n* = 715), Aklan (Region VI, *n* = 621), and Iloilo (Region VI, *n* = 570). Notably, three of the five most affected provinces—Capiz, Aklan, and Iloilo—are located within Region VI. Swine demography data from the FAO GLW dataset for the 2020 swine population (Figure 1A) were utilized to calculate standardized ASF outbreak reporting burden (Figure 1C). After accounting for swine population density, Oriental Mindoro maintained the highest disease burden at 89.37 per 10,000 pigs. The Dinagat Islands recorded the second-highest incidence (76.65 per 10,000 pigs), despite a lower absolute case count (*n* = 34). High standardized reporting burden were also observed in Occidental Mindoro and Aklan, which recorded 58.40 and 53.62 cases per 10,000 pigs, respectively, followed closely by Aurora at 52.31 cases per 10,000 pigs. On a broader scale, the spatial patterns observed in Figures 1B,C remain at a similar scale. A complete list of all affected provinces, including their respective swine populations and standardized reporting burden, is provided in Supplementary Table S1.

**Figure 1:**
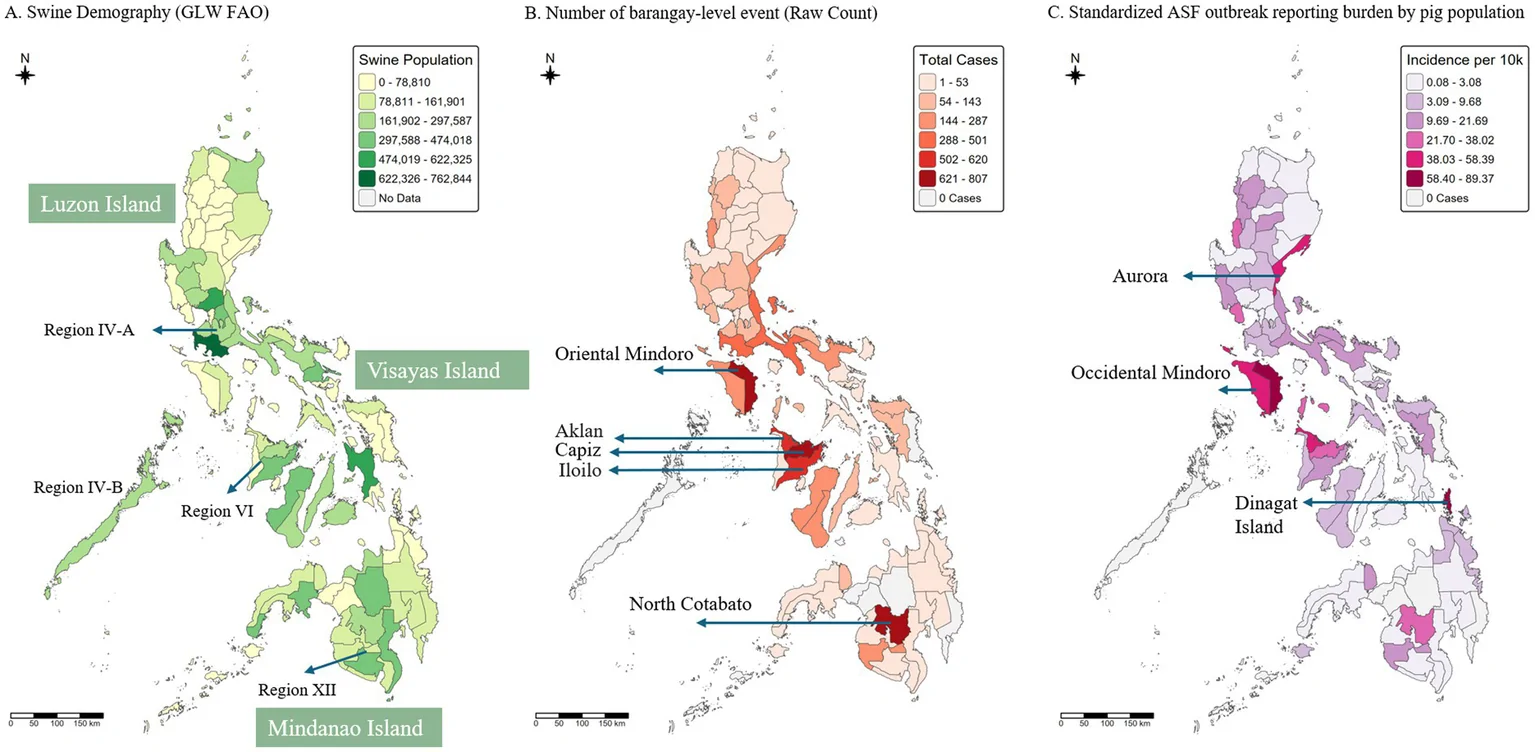
The spatial distribution of ASF varied across the 2022–2024 surveillance period. Baseline swine demography (GLW4) indicated that pig populations were concentrated in core provinces in Central Luzon, as well as in selected provinces in the Visayas and Mindanao, based on the 2020 map (Figure 1A). When mapping raw cumulative barangay-level event (Figure 1B), the highest absolute counts were observed in provinces with larger swine populations, including Iloilo (570 cases) and North Cotabato (715 cases), reflecting underlying population distribution. After adjusting for pig density to estimate standardized ASF outbreak reporting burden (Figure 1C), the spatial pattern slightly differed from that of raw counts. The standardized ASF outbreak reporting burden were higher in several geographically isolated or mid-density provinces, including Oriental Mindoro (89.37 cases per 10,000 swine), Dinagat Islands (76.65), and Occidental Mindoro (58.40). These patterns indicate variation in outbreak occurrence relative to baseline swine population size across provinces.

The Global Moran’s *I* test was utilized as the first spatial cluster indicator, yielding significant results (Moran’s Index = 0.199, *z* = 3.197, *p* < 0.001) at the national level and for each of the three major island groups—Luzon, Visayas, and Mindanao—using a Monte Carlo permutation test (nsim = 999). The temporal distribution of ASF events is visualized through a time series analysis at both the island group (Figure 2A) and regional levels (Figure 2B). The analysis identifies distinct temporal dynamics across the three major island groups. Luzon and Mindanao exhibited a relative increase in reports during the second half of 2024, specifically between July and October (October 2024 consist of partial-month data). Conversely, the Visayas followed a different trend, characterized by periodic fluctuations throughout the 2022–2023 period, reaching a recorded peak in June 2023.

**Figure 2:**
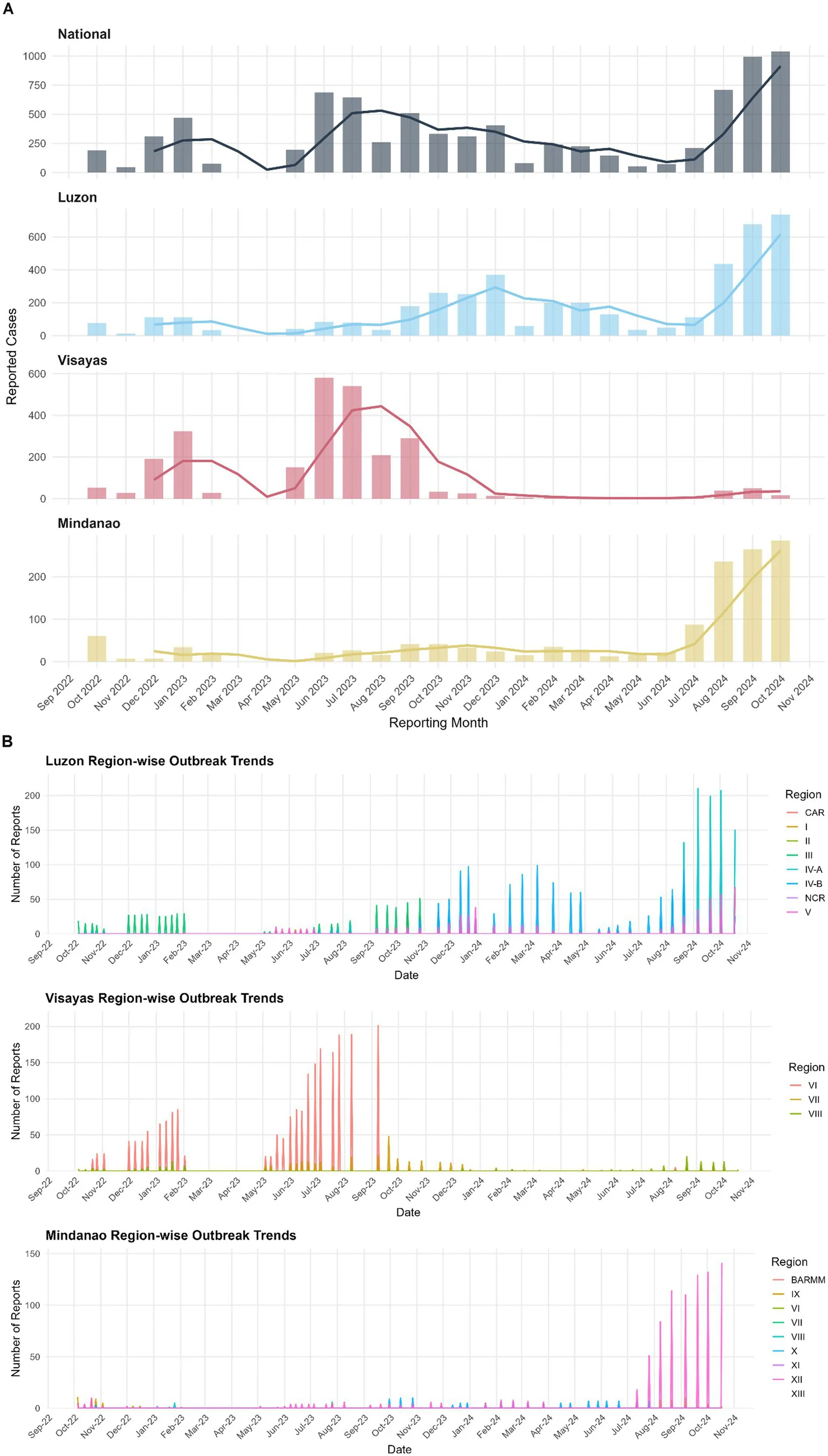
**(A)** National and island-level epidemic curves. Monthly outbreak cases ovelayed with a 3-month moving average trendiline. **(B)** Island level.

Following these results, a hot spot analysis using the Getis-Ord Gi* statistic was performed to identify statistically significant spatial clusters of ASF events. This analysis identified clusters within Regions VI, IV-B, and IV-A, where varying levels of red correspond to statistical confidence levels of 90, 95, and 99% (Figure 3). These results indicate areas with a high concentration of ASF occurrences.

**Figure 3:**
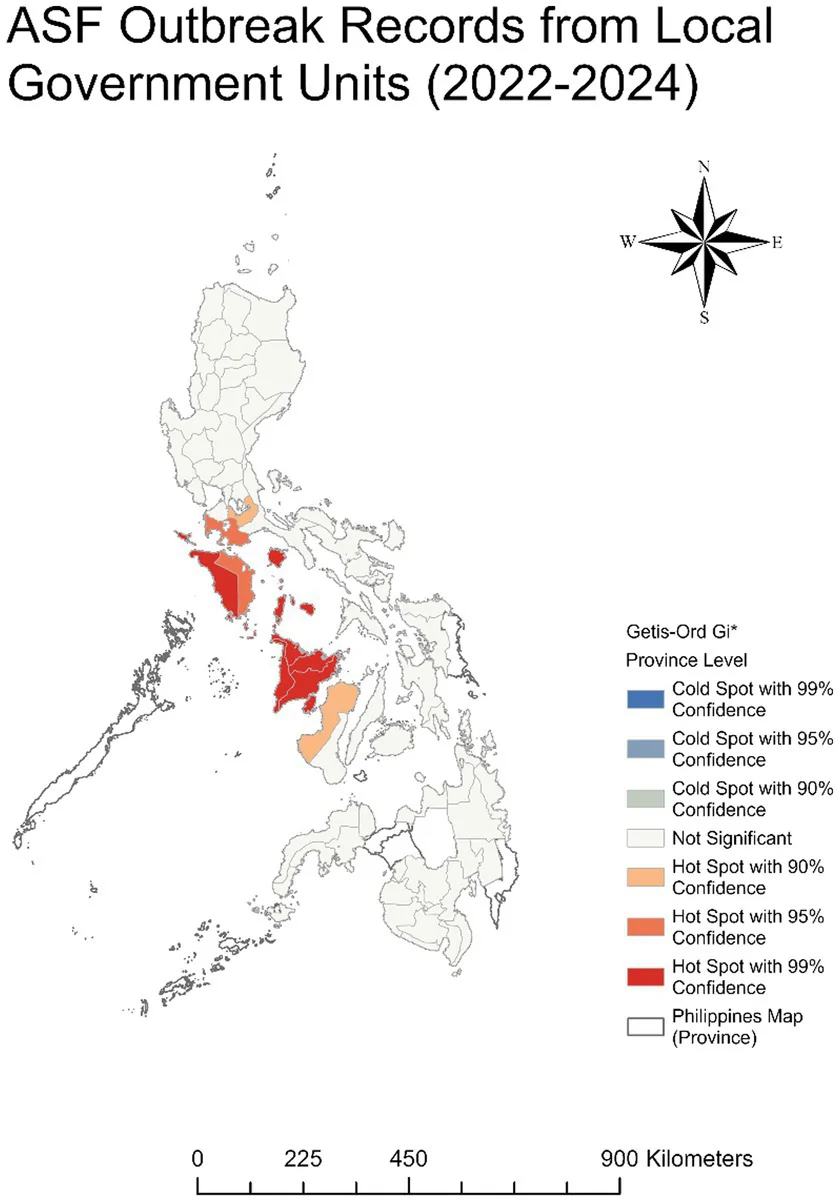
Hot spot analysis target in Region VI, Region IV-B and Region IV-A. Hot spot analysis (Getis-Ord Gi*).

A SaTScan space–time permutation model identified 12 statistically significant retrospective clusters (*p* < 0.001), with radii ranging from 7.13 to 183.2 km. The geographical distribution included five clusters in Luzon, four in the Visayas, and two in Mindanao. Additionally, one inter-island cluster was detected spanning the Visayas and Mindanao. These results indicate that while most ASF space–time clusters were localized, certain events exhibited spatial–temporal connectivity across maritime boundaries. Detailed cluster characteristics are provided in Table 1.

**Table 1:** Twelve spatiotemporal clusters identified in different main islands.

Cluster	Island	Radius (km)	Start date	End date	Duration (days)	Observed	Expected	Observed/expected
1	Luzon	122.28	2022-10-01	2023-03-31	181	310	75.19	4.12
2	Visayas	80.01	2022-10-01	2022-12-31	91	243	132.34	1.84
3	Visayas	30.69	2022-10-01	2022-12-31	91	25	3.6	6.95
4	Visayas	61.52	2023-04-01	2023-09-30	182	1,358	386.74	3.51
5	Luzon	7.13	2023-04-01	2024-06-30	456	44	5.91	7.44
6	Luzon	19.23	2023-07-01	2023-09-30	91	43	12.94	3.32
7	Luzon	49.72	2024-01-01	2024-06-30	181	508	73.11	6.95
8	Inter-island	142.34	2024-01-01	2024-06-30	181	90	22.01	4.09
9	Mindanao	7.31	2024-01-01	2024-03-31	90	34	4.32	7.87
10	Visayas	183.2	2024-04-01	2024-09-30	182	201	114.4	1.76
11	Luzon	67.14	2024-07-01	2024-12-31ª	183ª	762	292.12	2.61
12	Mindanao	138.4	2024-07-01	2024-09-30	91	510	189.37	2.69

± Cluster Order is according to its Start Date; Number of computing replications: 999.

aOctober 2022 and October 2024 consist of partial-month data.

^*^p < 0.00001 for all 12 clusters.

## Discussion

This study provides an updated epidemiological overview of ASF in the Philippines from October 2022 to October 2024, examining spatial, temporal, and space–time cluster patterns at national, three main islands, and regional levels. Utilizing longitudinal surveillance data from the National African Swine Fever Prevention and Control Program (NASFPCP), the analysis identifies a substantial shift in the distribution of the disease following the implementation of government intervention and elimination policies.

While previous research covering the initial 2019–2022 outbreak period identified mainland Luzon as the primary epicenter with notable seasonality using 19,697 farm-level outbreaks concentrated in Central Luzon clusters (12), the current findings indicate a geographical shift toward the central island groups. Although Luzon accounted for 52.10% of total reports in present research, this proportion largely reflects its high swine population density. When adjusting for host population at risk, the highest reporting burden were concentrated in separate island provinces rather than the main archipelagic landmasses. Specifically, the highest burdens occurred in Oriental Mindoro (89.37 cases per 10,000 swine), the Dinagat Islands (76.65 cases per 10,000 swine), and Occidental Mindoro (58.40 cases per 10,000 swine). Consequently, Mindoro, the seventh-largest island in the Philippines, experienced a substantial disease burden during the 2022–2024 surveillance period.

Spatial descriptive statistics (Figure 1) indicated that the Visayas, particularly the Western Visayas (Region VI) provinces of Capiz, Aklan, and Iloilo, experienced more frequent ASF outbreaks between 2022 and 2024. Space–time permutation analysis (Table 1) further identified two major outbreak clusters in Region VI: an initial cluster (Cluster #2) in late 2022 (October–December) and a subsequent cluster (Cluster #4) from April to September 2023. The spatial distribution observed in this study, particularly the concentration of outbreaks in island provinces like Mindoro (Region IV-B) and the hotspot analysis using the Getis-Ord Gi* statistic identified Western Visayas (Region VI) as a high-risk zone over the study period (Figure 3), suggests that natural geographical barriers were insufficient to prevent significant ASF transmission. In addition, the repeated occurrence of outbreaks in these geographic areas implies either persistent local viral circulation or frequent re-introduction via inter-provincial pig movements. Oriental Mindoro and the clusters of provinces in Western Visayas (Capiz, Aklan, and Iloilo) emerged as the primary hotspots. The occurrence of high-intensity outbreaks in provinces isolated from the major landmasses of Luzon and Mindanao suggests that maritime borders do not completely block viral spread. Furthermore, the detection of inter-island space–time clusters (Cluster #8, Table 1) warrants further exploration regarding the hypothesis that maritime trade and the movement of contaminated pork products across sea routes could play a more substantial role in transmission than contiguous land-based spread in these regions. While our current analysis focuses on spatiotemporal patterns, these findings highlight a potential pathway that merits future investigation integrating movement-related variables.

A plausible alternative explanation for this shift involves the demographic restructuring of the Philippine swine industry following the ASF outbreak wave. The observed temporal patterns across Luzon, the Visayas, and Mindanao appear closely linked to pig population dynamics—specifically turnover and repopulation following required depopulation measures. Between 2019 and 2022, the major swine hubs in Luzon were severely impacted (6, 12), leading to total depopulation and the enforcement of intensive surveillance policies. Consequently, swine production underwent a major industry shift southward to the Visayas and Mindanao. This resulted in these regions hosting not only larger pig populations but also a high proportion of immunologically naïve, susceptible animals. For example, extensive depopulation in Western Visayas between June and September 2023 led to a temporary reduction in susceptible pigs and a period of apparent epidemiological silence. However, as restocking gradually resumed, the expanding susceptible population might provide conditions favorable for ASF re-emergence several months later. This cyclical pattern of depopulation, restocking, and reinfection has been documented in other ASF-endemic countries (23, 24) and represents a potential challenge for sustained disease control. Therefore, during the 2022–2024 study period, these southern regions became more vulnerable as increased production activities and commercial trade intensified the risk of virus introduction. Routinely collecting data on restocking, pig movements, and transport logistics could substantially enhance future outbreak investigation and tracking efforts.

Beyond these spatial shifts, temporal analysis indicates that the Philippine ASF epidemic consists of regionally distinct waves rather than synchronized events (Figure 2A). Nationally, the epidemic curve exhibited three major waves. The first peaked in January 2023 with 470 cases, driven primarily by the Visayas (324 cases, 68.9%). The second wave in 2023 followed a bimodal pattern: peaks in June (687 cases) and July (646 cases) were predominantly associated with the Visayas (84.6% of June cases), while a subsequent surge from September to December was driven by Luzon, peaking at 369 cases in December. The third and largest wave occurred in late 2024, reaching 1,038 cases in October; this was driven by concurrent spikes in Luzon (736 cases, 70.9%) and Mindanao (285 cases, 27.5%), whereas the Visayas reported only 17 cases. Overall, the Visayas contributed substantially to the 2023 epidemic dynamics, whereas Luzon and Mindanao predominated during the 2024 surge.

The divergence between absolute case counts and swine population-adjusted standardized ASF outbreak reporting burden, coupled with the staggered regional waves across different major islands—such as 2023 surge in Region IV-B and the late 2024 peak in Mindanao’s Region XII—highlights a complex and non-uniform ASF epidemic trajectory (Figure 2B). As illustrated, transmission dynamics exhibit significant spatial and temporal heterogeneity: while some regions experienced recurrent, high-intensity outbreaks throughout multiple seasons, others remained relatively quiescent, such as Regions VII and VIII in the Visayas, or showed only sporadic reports during the study period. Table 1 and the space–time clusters in Supplementary Material S2 provide additional resolution, capturing the granular details of these shifting transmission dynamics. Although the observational study design cannot establish definitive causality, these asynchronous patterns likely reflect regional variations in pig production systems—specifically the differing proportions of commercial versus smallholder farms—as well as distinct local risk factors (25) and the varying implementation of control policies. Furthermore, our findings suggest that these dynamics may be influenced by localized livestock movement, supply chain logistics, and regional environmental factors. While these remain hypotheses that warrant further investigation due to empirical data limitations, our results indicate that national-level summaries may obscure critical sub-national transmission dynamics, thereby necessitating region-specific surveillance and intervention strategies.

While smallholder or backyard pig farms accounted for approximately 76.5% of the swine population in the Philippines as of 2023, according to Philippine Statistic Authority, regional disparities influence ASF diagnostic and surveillance. The Visayas, characterized by numerous islands and a higher proportion of smallholder farmers, faces unique challenges that may lead to underreporting and delayed detection compared to Luzon, which benefits from more diagnostic laboratories and centralized governmental resources (26, 27). The fragmented geography, limited biosecurity, and restricted veterinary access in Visayas complicate routine surveillance, emphasizing the need for targeted policy interventions/surveillance to enhance ASF outbreak detection and management of animal movements in these ASF hotspot areas.

Comparatively, community-driven local approaches, such as the participatory action model employed in Northern Uganda (28) could offer valuable lessons in the context. These strategies foster local engagement and shared responsibility and ownership, which can improve ASF control sustainability in regions dominated by smallholder and/or backyard farming systems. Adapting similar models could enhance hog producer compliance and resilience in Philippine locales identified as persistent ASF hotspots. In this regard, our study establishes a critical spatial baseline that serves as a foundational reference for evidence-based policy and intervention. By identifying high-risk geographic clusters in different administrative units, this baseline allows for the strategic optimization of vaccine deployment and provides a quantitative benchmark to rigorously evaluate the efficacy of future ASF control programs.

The Philippine Department of Agriculture has allocated ₱240 million (~$4 million USD) to procure 500,000 doses of the ASF live-attenuated vaccine (AVAC ASF LIVE vaccine). This funding also covers vaccination equipment, cold storage facilities, test kits, and logistics (29). This large-scale procurement and the push for implementation underscore the urgent need for this epidemiological study. A comprehensive understanding of the pre-vaccination ASF landscape is critical prior to initiating a nationwide mass vaccine rollout. While vaccines are not a panacea for ASF and concerns regarding efficacy persist, the evolving vaccination landscape has sparked considerable debate regarding alignment with international benchmarks. Notably, in May 2025, WOAH established the first international standards for ASF vaccines at its 92nd General Session, emphasizing the necessity of a high-quality, safe, and robust regulatory framework. Furthermore, the emergence of novel recombinant Genotype I/II ASFV strains across the Asia-Pacific region (particularly in China, Vietnam, and Russia (30–32)) presents a significant threat to the Philippines. Current evidence suggests that existing ASF vaccines lack cross-protection against Genotype I/II recombinant (33), raising substantial concerns. Although these strains have not yet been detected domestically in the Philippines, the risk of introduction necessitates continuous genomic surveillance and rigorous epidemiological monitoring to ensure early detection and rapid containment of potential future outbreaks.

This study has several limitations. First, our reliance on ASF barangay-level data aggregation is susceptible to the Modifiable Areal Unit Problem (MAUP), where spatial patterns and cluster detection may be sensitive to the arbitrary nature of administrative units. Consequently, our findings represent epidemiological trends at this specific spatial scale, and results may vary if analyzed at more granular levels (e.g., the farm level or the individual pig level). However, since a high proportion of farms outside Luzon islands are smallholder or semi-commercial operations with relatively homogeneous production characteristics (typically 20 head or fewer) (34, 35), barangay-level data provide a reasonable approximation of regional outbreak intensity in most provinces. Second, FAO GLW4 data serves only as an approximate 2020 baseline, actual swine populations may have varied during the study period due to ASF-driven culling and restocking; nevertheless, it remains the most robust data source for estimating swine distributions. Finally, although underreporting is an inherent challenge in passive surveillance, ASF is a WOAH-notifiable disease, and ongoing national surveillance efforts support the overall validity of the observed epidemiological trends. These findings thus offer insights into the current status of ASF epidemiology in the Philippines prior to the implementation of national vaccination programs.

In conclusion, our research highlights key regions—specifically Regions IV-A, IV-B, VI, and XII—where persistent ASF outbreaks warrant prioritized government attention and resource allocation to ensure sustainable intervention. Although the timeline for official ASF vaccine approval remains uncertain, these foundational epidemiological insights from 2022 to 2024 provide the evidence-based framework necessary to support future vaccination strategies and broader ASF control efforts.

## Data Availability

The original contributions presented in this study are included in the article and/or its Supplementary Material. The data used in this study are the property of the Government of the Philippines and were shared with the research team for the purposes of conducting this study and supporting training activities in the country. Requests for access to the underlying data should be directed to the Philippine Bureau of Animal Industry and the International Training Center on Pig Husbandry of the Philippines. Further inquiries can be directed to the corresponding author.
